# PRGF Membrane with Tailored Optical Properties Preserves the Cytoprotective Effect of Plasma Rich in Growth Factors: In Vitro Model of Retinal Pigment Epithelial Cells

**DOI:** 10.3390/ijms241311195

**Published:** 2023-07-07

**Authors:** Eduardo Anitua, Francisco Muruzabal, María de la Fuente, Susana Del Olmo-Aguado, Mohammad H. Alkhraisat, Jesús Merayo-Lloves

**Affiliations:** 1 BTI—Biotechnology Institute, 01007 Vitoria, Spain; francisco.muruzabal@bti-implant.es (F.M.); maria.delafuente@bti-implant.es (M.d.l.F.); mohammad.hamdan@bti-implant.es (M.H.A.); 2 University Institute for Regenerative Medicine and Oral Implantology—UIRMI (UPV/EHU-Fundación Eduardo Anitua), 01007 Vitoria, Spain; 3 Fundación de Investigación Oftalmológica, Instituto Oftalmológico Fernández-Vega, 33012 Oviedo, Spain; solmo@fio.as (S.D.O.-A.); merayo@fio.as (J.M.-L.); 4 Instituto de Investigación Sanitaria del Principado de Asturias (ISPA), 33011 Oviedo, Spain

**Keywords:** platelet rich plasma, PRGF, oxidative stress, age-related macular degeneration, AMD, retinal pigment epithelial cells, transparence, growth factors

## Abstract

The present study evaluates the ability of a novel plasma rich in growth factors (PRGF) membrane with improved optical properties to reduce oxidative stress in retinal pigment epithelial cells (ARPE-19 cells) exposed to blue light. PRGF was obtained from three healthy donors and divided into four main groups: (i) PRGF membrane (M-PRGF), (ii) PRGF supernatant (S-PRGF), (iii) platelet-poor plasma (PPP) membrane diluted 50% with S-PRGF (M-PPP 50%), and (iv) M-PPP 50% supernatant (S-PPP 50%). ARPE-19 cells were exposed to blue light and then incubated with the different PRGF-derived formulations or control for 24 and 48 h under blue light exposure. Mitochondrial and cell viability, reactive oxygen species (ROS) production, and heme oxygenase-1 (HO-1) and ZO-1 expression were evaluated. Mitochondrial viability and cell survival were significantly increased after treatment with the different PRGF-derived formulations. ROS synthesis and HO-1 expression were significantly reduced after cell treatment with any of the PRGF-derived formulations. Furthermore, the different PRGF-derived formulations significantly increased ZO-1 expression in ARPE-19 exposed to blue light. The new PRGF membrane with improved optical properties and its supernatant (M-PPP 50% and S-PPP 50%) protected and reversed blue light-induced oxidative stress in ARPE-19 cells at levels like those of a natural PRGF membrane and its supernatant.

## 1. Introduction

In 2020, different vitreoretinal diseases such as glaucoma, age-related macular degeneration (AMD), diabetic retinopathy, and retinitis pigmentosa contributed to more than 6 million adults over the age of 50 being blind and more than 13 million adults over the age of 50 suffering from moderate to severe vision loss [1,2].

The etiopathogenesis of most of these pathologies has been associated with inflammatory and oxidative stress response mechanisms [3]. Although no effective therapy has been developed today for the treatment of any of these diseases, systemic antioxidant supplementation has been included as a standard treatment for the management of all these retinal diseases in order to delay their progression [4].

However, the problem for the treatment of vitreoretinal diseases is not only the lack of therapies, but also the need for an effective and efficient therapeutic route of application. There are several options for the application of different therapies in the treatment of retinal diseases, such as topical administration, intravitreal, periocular, subconjunctival, or retrobulbar injection, and systemic administration, among others, but each has its advantages and disadvantages [5].

The most common drawbacks are the bioavailability of treatments at the back of the eye when applied topically or systemically due to the biological barriers that make up the eye, and the invasiveness of other routes of application such as intravitreal or subretinal, among others, which favor drug bioavailability but carry several associated risks such as retinal detachment, cataracts or endophthalmitis [6]. In addition, intravitreal or intraocular tissue injection of the drug has a short bioavailability time, and several visits to the ophthalmologist are required to apply the treatment under sterile conditions, as in the case of intraocular injection of anti-VEGF for the treatment of wet AMD [7].

With the aim of optimizing the availability of therapeutic drugs for the treatment of diseases of the back of the eye and to reduce the number of administrations and thus the risk of suffering from some associated ocular disorders, there has been a great deal of research effort in nanomedicine to improve drug delivery to the posterior segment of the eye [5]. However, these delivery systems should possess certain characteristics such as being biodegradable, biocompatible, stable in biological environments, and controlled release, among others, to be suitable for the treatment of vitreoretinal pathologies [5]. Despite the advantages of using resorbable inserts, some patients report discomfort due to foreign body detection, blurred vision, and ocular irritation [8]. Although advances in the field of ocular drug delivery have been enormous, the search for platforms that combine controlled and painless delivery performance while maintaining visual capability is still ongoing.

In the last two decades, plasma rich in growth factors (PRGF) technology has positioned itself as a very interesting therapy in the ophthalmological field [9,10,11,12]. Several factors have attracted attention to PRGF. First, it is a pure platelet-rich plasma (no leukocytes) with anti-inflammatory and anti-fibrotic properties [13,14,15,16]. Second, it is a highly versatile platform, as several formulations can be obtained such as eye drops, an injectable formulation, a membrane, or a clot [17]. After platelet activation, a fibrin scaffold with a high content of bioactive proteins from plasma and platelets is obtained. This generates a dynamic system that assures the localization of PRGF’s bioactive molecules and their progressive release at the injury site [18]. However, the release of PRGF content could be forced by squeezing the PRGF membrane to obtain the eye drop formulation [19]. Both eye drops and membranes have been widely used for the treatment of different ocular surface pathologies [12,20,21,22]. In recent studies, both membrane and injectable formulations obtained from PRGF technology have been used for the treatment of macular holes, paving the way for the treatment of other vitreoretinal diseases [23,24,25]. An interesting effect of PRGF in this context is its ability to reduce oxidative stress and to improve the survival of retinal pigment epithelial cells exposed to blue light [26,27,28,29].

The development of a new formulation of the PRGF should not jeopardize its biological effects. In a recent study, a PRGF membrane with improved optical properties has been tailored for use on the ocular surface [30]. This more transparent membrane could be used as a drug delivery system for the treatment of vitreoretinal diseases without hindering the patient’s vision. However, there is a need for assessing its suitability for eye fundus.

For that, the main objective of the present study has been to analyze the efficacy of the new PRGF membrane with improved optical properties and its supernatant in reducing oxidative stress induced by blue light exposure in retinal pigment epithelial cells compared to the native PRGF membrane and its supernatant.

## 2. Results

### 2.1. Blood-Derived Preparations

Platelets and leukocytes were measured in the peripheral blood of the three donors before obtaining the different formulations tested in the present study; the mean ± SD values were 235 ± 116 × 10^6^/mL and 7.0 ± 0.9 × 10^6^/mL, respectively. Platelet and leukocyte values in the whole plasma column collected after centrifugation were 471 ± 206 and 0.2 ± 0.1, respectively. The mean platelet enrichment of the PRGF preparations over the baseline concentration of platelets in whole blood was 2.0-fold.

### 2.2. Protection Assay

**Figure 1:**
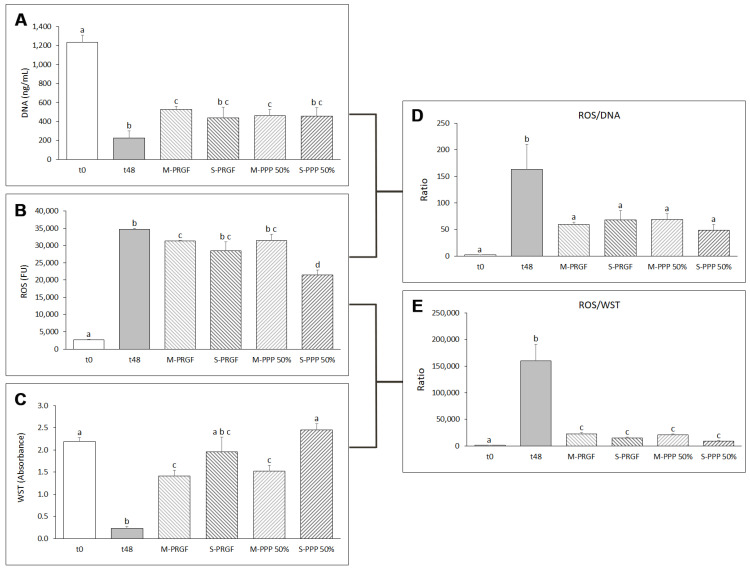
Evaluation of the different blood-derived formulations in ARPE-19 cells in the protection assay. Cell survival as measured by DNA concentration (**A**), ROS production (**B**), mitochondrial viability measured by WST-1 (**C**), ratio of ROS expression to cell number (ROS/DNA) (**D**), and ratio of ROS expression to mitochondrial viability (ROS/WST) (**E**). Treatment groups that do not share at least one letter are statistically significant at *p* < 0.05.

After incubating ARPE-19 cells with blue light for 48 h while treating them with the different formulations developed in the present study (M-PRGF, S-PRGF, M-PPP 50%, and S-PPP 50%), several measurements were performed on these cells such as DNA concentration, mitochondrial viability, and reactive oxygen species (ROS) synthesis. DNA concentration results obtained by the CyQUANT assay showed that the influence of blue light significantly reduced the DNA concentration after 48 h of exposure (Figure 1A), indicating that the number of cells is significantly reduced after being treated with blue light for 48 h. However, treatment with the different formulations mitigated the reduction in cell number and thus cell death during exposure to blue light for 48 h. Cell number, represented as DNA concentration, was higher in all blood-derived formulations analyzed in the present study compared to the 48-h control group (t48), reaching statistical significance when cells were treated with the membrane formulations (M-PRGF and M-PPP 50%) (Figure 1A).

The effect of blue light exposure on mitochondrial activity was measured as absorbance using the WST-1 reagent. Except for S-PRGF, the different PRGF-derived formulations significantly (*p* < 0.05) increased WST-1 absorbance values after 48 h of blue light exposure compared to the control treatment (t48) (Figure 1C). Furthermore, cells treated with S-PRGF and S-PPP 50% showed WST-1 levels similar to the basal state of the cells (t0). In addition, cells treated with S-PPP 50% showed a significant increase in WST-1 absorbance values with respect to ARPE-19 cells treated with the membrane formulations (M-PRGF and M-PPP 50%).

ROS levels were also measured to assess the protective effect of the different blood-derived formulations tested in this study on ARPE-19 cells exposed to blue LED light for 48 h. The results showed that ROS expression of ARPE-19 cells treated with the different PRGF-derived formulations was reduced with respect to the 48-h control group (t48), showing significant differences of M-PRGF and S-PPP 50% regarding t48 (Figure 1B). In addition, S-PPP 50% significantly (*p* < 0.05) reduced ROS levels compared to the other plasma formulations. In all cases, significant differences in ROS production were observed between cells treated with PRGF and PPP 50% formulations and their initial state (t0).

In addition, ROS production was evaluated in relation to the number of cells present in each well (ROS/DNA) (Figure 1D) and mitochondrial activity (ROS/WST-1) (Figure 1E). The results revealed that the levels of ROS produced by each cell treated with either PRGF or PPP 50% formulations were similar to the basal state (t0) (*p* < 0.05) and showed statistically significant differences (*p* < 0.05) with respect to the control group incubated for 48 h with blue light (t48) (Figure 1D). Furthermore, no differences (*p* > 0.05) were observed in the ROS/DNA ratio between the different PRGF and PPP 50% formulations. Similar results were found when ROS levels were related to the mitochondrial activity of cells (WST) treated with the different blood-derived formulations, where both formulations (membrane and supernatant) obtained from PRGF and PPP 50% significantly (*p* < 0.05) reduced ROS levels with respect to t48 when related to cellular mitochondrial activity (ROS/WST-1) (see Figure 1E). However, the ROS/WST-1 levels of cells treated with the different plasma formulations were significantly higher (*p* < 0.05) compared to the initial state (t0).

**Figure 2:**
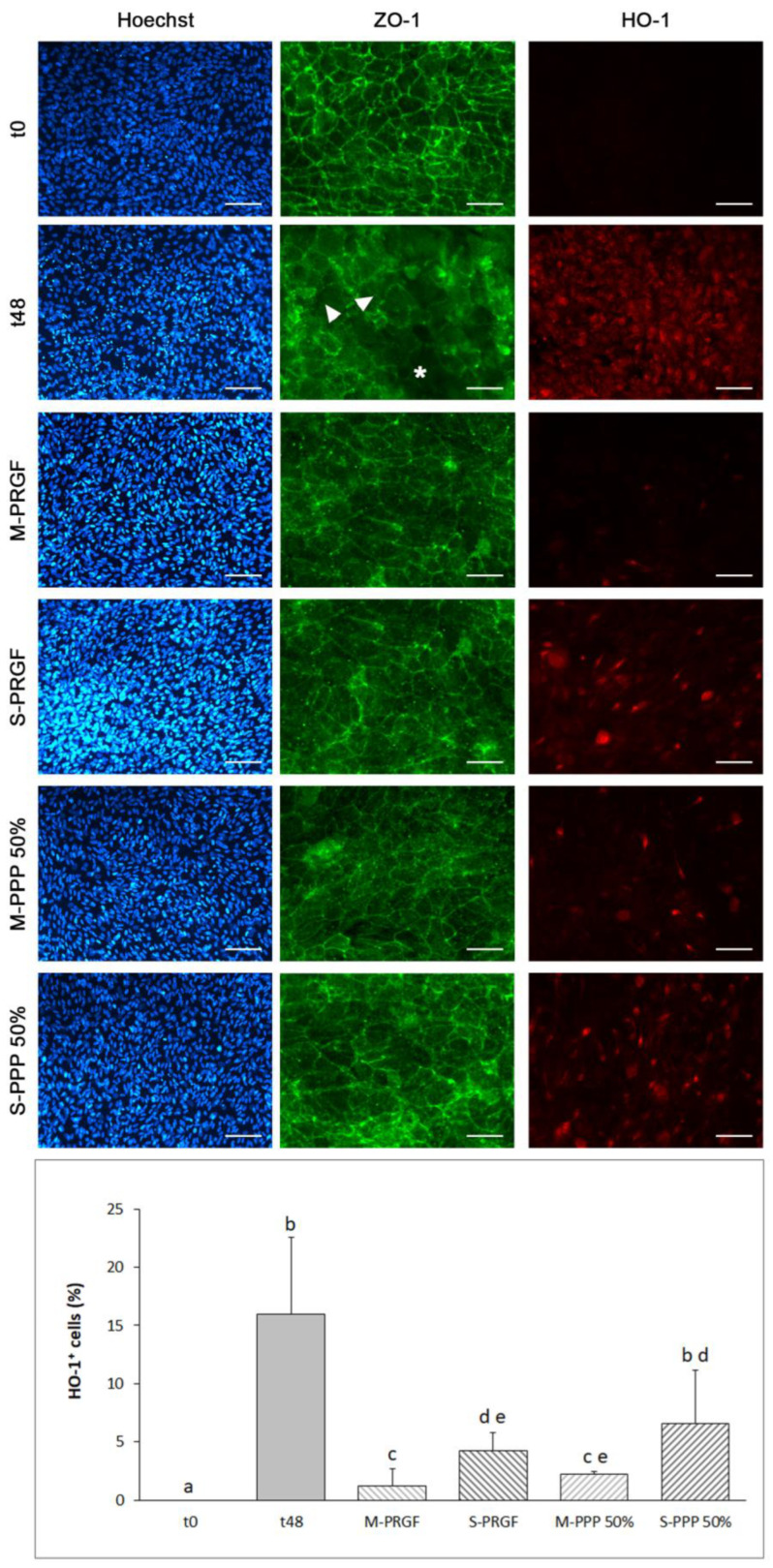
Immunofluorescence expression of heme oxygenase 1 (HO-1) and ZO-1 in ARPE-19 cells used in the protection assay. Representative immunofluorescence images of Hoechst 33342, HO-1, and ZO-1 in ARPE-19 cells at t0 and after incubation with the different treatments (M-PRGF, S-PRGF, M-PPP 50%, and S-PPP 50%) and control (t48) for 48 h while maintaining exposure to blue light. ARPE-19 cells showed continuous labeling of the cell membrane for ZO-1 immunofluorescence at basal state (t0). After exposure of ARPE-19 cells to blue light for 48 h (t48), a marked loss of ZO-1 staining was observed at the periphery of the cells (white arrowhead), increasing its cytoplasmic localization. In addition, ARPE-19 cell-free spaces were observed, suggesting a partial loss of cells after exposure to blue light for 48 h (asterisk). Treatment of ARPE-19 with the different plasma-derived formulations reduced the delocalization of ZO-1 staining after 48 h of blue light treatment. HO-1 expression was significantly increased after 48 h of exposure to blue light. The different blood-derived formulations reduced the expression of HO-1 compared to the control group t48. Treatment groups that do not share at least one letter are statistically significant at *p* < 0.05. Scale bar for Hoechst and HO-1 images: 200 µm; and for ZO-1 images: 800 µm.

Heme oxygenase-1 and ZO-1 expression was evaluated in ARPE-19 cells exposed to blue light for 48 h (Figure 2); cell nuclei were also stained with Hoechst 3342. Figure 2 shows representative images of the different immunofluorescence stains (Hoechst, HO-1 and ZO-1) obtained for each sample analyzed in the protection assay performed in this study. Immunofluorescence for HO-1 showed that, except for the S-PPP 50% formulation, the rest of the PRGF and PPP 50% formulations significantly reduced (*p* < 0.05) the percentage of HO-1 positive cells with respect to the control group at 48 h of blue light exposure. In addition, the percentage of cells expressing HO-1 increased significantly (*p* < 0.05) after exposure to blue light for 48 h and subsequent treatment with either of the PRGF-derived formulations compared to t0. Both membrane preparations (M-PRGF and M-PPP 50%) reduced the number of cells that were HO-1 positive regarding the supernatant preparation, reaching statistical differences (*p* < 0.05) between M-PRGF and both supernatant preparations (S-PRGF and S-PPP 50%) and between M-PPP 50% and S-PPP 50% but not regarding S-PRGF (see Figure 2).

Immunostaining of zonula occludens-1 (ZO-1) showed continuous labeling in the cell–cell contact regions at baseline (t0). However, when cells were exposed to blue light for 48 h (t48), a marked loss of ZO-1 staining was observed at the periphery of the cells, increasing its cytoplasmic localization (Figure 2). In addition, in the t48 group, empty spaces surrounded by several ARPE-19 cells stained discontinuously with ZO-1 were observed, suggesting that some cells had been displaced from the monolayer. On the other hand, the different blood-derived formulations reduced the delocalization of ZO-1 staining in ARPE-19 cells exposed to blue light for 48 h, showing a slight reduction of ZO-1 staining compared to t0. No cell loss was observed in any sample treated with any of the formulations (M-PRGF, S-PRGF, M-PPP 50%, or S-PPP 50%) in contrast to the results observed in the t48 group.

### 2.3. Reversion Assay

**Figure 3:**
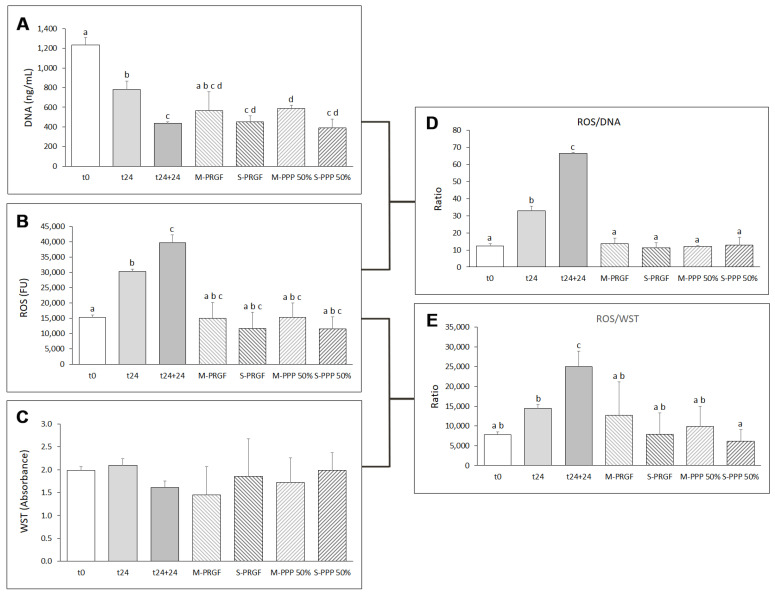
Evaluation of the different blood-derived formulations in ARPE -19 cells in the reversion assay. Cell survival as measured by DNA concentration (**A**), ROS production (**B**), mitochondrial viability as determined by WST-1 (**C**), ratio of ROS expression to the number of cells (ROS/DNA) (**D**), and ratio of ROS expression to mitochondrial viability (ROS/WST) (**E**). Treatment groups that do not share at least one letter are statistically significant at *p* < 0.05.

Exposure of ARPE-19 cells to blue light for 24 h (t24) showed a significant reduction in cell number from the initial time (t0). This significant decrease in cell number was maintained when cells were exposed to blue light for an additional 24 h (t24 + 24) (Figure 3A). Treatment of cells with the membrane preparation of both blood-derived formulations (M-PRGF and M-PPP 50%) dampened the decrease in cell number after the first 24 h of incubation with blue light. M-PRGF did not show significant differences (*p* > 0.05) in cell number with respect to the control groups at any of the blue light exposure times (t0, t24 or t24 + 24); however, cells treated with M-PPP 50% suffered a significant reduction in cell number with respect to t24, but their decrease was not as drastic as in the t24 + 24 group, showing significant differences in cell number between both treatment groups (M-PPP 50% and t24 + 24).

In contrast, a significant reduction in cell number was observed when cells were treated with the supernatant preparation of both types of formulation (S-PRGF and S-PPP 50%), showing cell levels analogous to that of the t24 + 24 control group (Figure 3A). No significant differences (*p* > 0.05) in cell number were observed between the different formulations developed in the present study (M-PRGF, S-PRGF, M-PPP 50%, and S-PPP 50%).

The impact of blue LED lights on ARPE-19 cells was analyzed by ROS production after the initial 24 h of blue light exposure (t24). ROS levels increased significantly after the first 24 h of exposure to blue light compared to basal ROS synthesis (t0) (Figure 3B). Afterward, cells cultured with any of the blood-derived formulations developed in the present study (M-PRGF, S-PRGF, M-PPP 50%, and S-PPP 50%) showed a marked reduction in intracellular ROS production after 24 h of treatment compared to cells treated with blue light for 24 h (t24) and 48 h (t24 + 24), exhibiting levels of ROS production similar to the basal state (t0) (Figure 3B). However, no significant differences (*p* < 0.05) were observed between the different blood-derived formulations and the different control groups (t0, t24, and t24 + 24). Nonetheless, when ROS production was evaluated in relation to the number of cells present in each well (ROS/DNA) (Figure 3D), the results showed that any blood-derived preparation (membrane and supernatant) in any of its formulations (PRGF or PPP-50%) significantly reduced (*p* < 0.05) the levels of ROS produced by the cells after 24 h of blue light treatment (t24) to levels similar to those of the basal state of the cells (t0).

On the other hand, mitochondrial activity assessed with WST-1 reagent in cells used in the reversion assay showed that no significant differences (*p* > 0.05) were observed between cells treated with any of the blood-derived formulations and cells in the basal state (t0) or cells exposed to blue LED light for 24 (t24) or 48 h (t24 + 24). However, when ROS production was assessed in relation to mitochondrial activity (ROS/WST-1) (Figure 3E), the different blood-derived formulations significantly (*p* < 0.05) reduced ROS/WST-1 levels with respect to t24 + 24 (Figure 3E), reaching ROS/WST-1 levels comparable to the basal state (t0).

**Figure 4:**
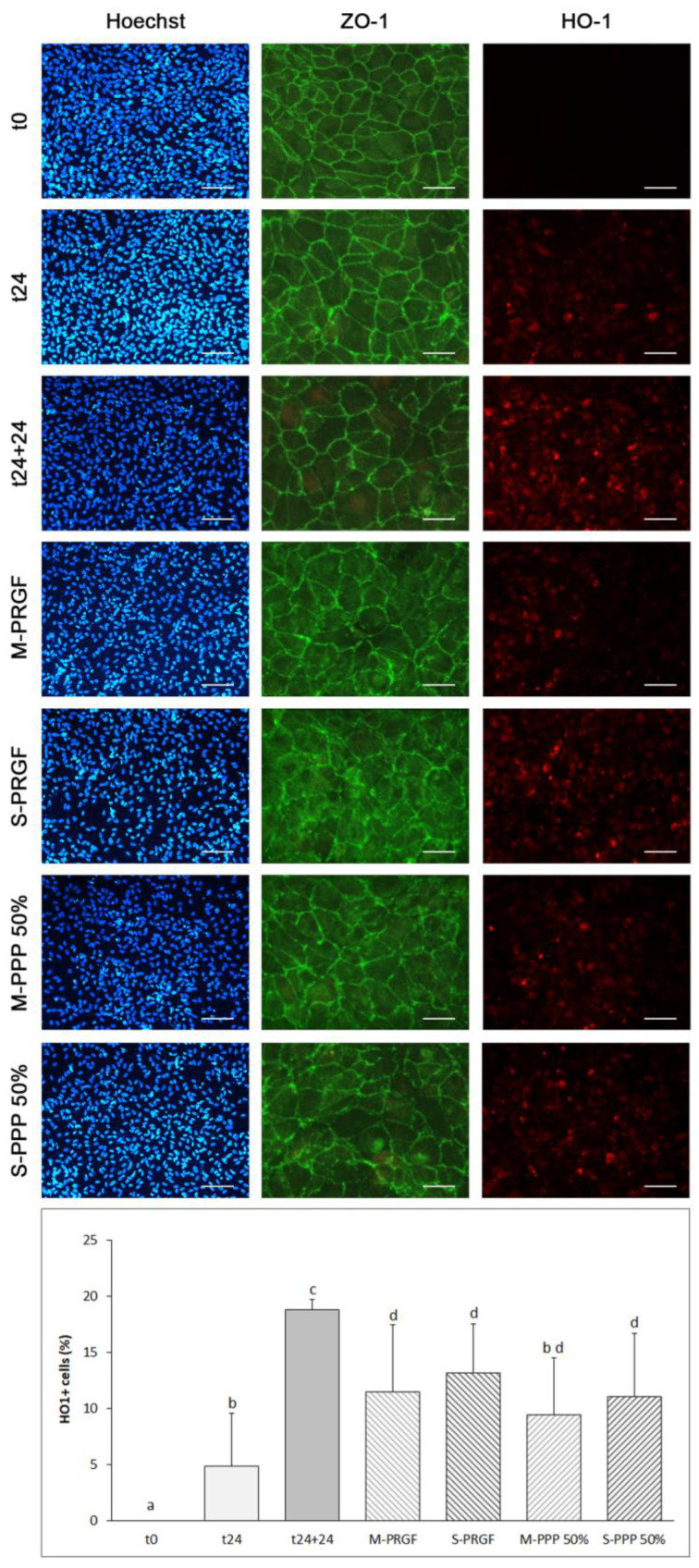
Immunofluorescence expression of heme oxygenase 1 (HO-1) and ZO-1 by ARPE-19 cells used in the reversion assay. Representative immunofluorescence images of Hoechst 33342, HO-1, and ZO-1 in ARPE-19 cells at t0, after being exposed for 24 h to blue light (t24) and then incubated with the different treatments (M-PRGF, S-PRGF, M-PPP 50%, and S-PPP 50%) and control (t24 + 24) for another 24 h while exposed to blue light. Intense ZO-1 immunostaining was observed at the cell membrane of ARPE-19 cells at basal state (t0) and after exposure to blue light for 24 h (t24). However, ZO-1 immunofluorescence was reduced at the cell periphery after exposure for another 24 h to blue light (t24 + 24), showing, at the same time, an increase in cytoplasmic ZO-1 immunostaining. The different blood-derived formulations increased ZO-1 immunostaining in the membrane of ARPE-19 cells compared to cells in the t24 + 24 group; however, cytoplasmic ZO-1 staining was also increased in cells treated with any of the blood-derived formulations compared to the control groups. As for HO-1 immunostaining, each of the blood-derived formulations (M-PRGF, S-PRGF, M-PPP 50%, and S-PPP 50%) reduced HO-1 expression after exposure to blue light for an additional 24 h (t24 + 24). Furthermore, M-PPP 50% maintained HO-1 expression in ARPE-19 cells regarding the t24 group. Treatment groups that do not share at least one letter are statistically significant at *p* < 0.05. Scale bar for Hoechst and HO-1 images: 200 µm; and for ZO-1 images: 800 µm.

Heme oxygenase-1 (HO-1) and ZO-1 expression was also assessed by immunofluorescence in the ARPE-19 cells used in the reversion assay (Figure 4). Figure 4 shows representative images of the different immunofluorescence stains (Hoechst, HO-1 and ZO-1) obtained for each sample analyzed in the reversion assay performed in the present study. Immunofluorescence for HO-1 showed that each of the blood-derived formulations (M-PRGF, S-PRGF, M-PPP 50%, and S-PPP 50%) dampened HO-1 expression after exposure to blue light for an additional 24 h, showing significant differences with respect to the control (t24 + 24). In addition, cells treated with M-PPP 50% maintained HO-1 expression compared to baseline (t24).

Immunostaining of zonula occludens-1 (ZO-1) cells exposed for 24 h to blue LED light showed similar continuous staining in cell–cell regions as in the basal situation (t0). Apparently, no loss of ZO-1 immunostaining was observed after 24 h of blue light exposure (Figure 4). However, when the cells were exposed to blue light for an additional 24 h (t24 + 24), a marked loss of ZO-1 staining was observed at the periphery of the cells with increasing cytoplasmic delocalization (Figure 4), as observed in the protection assay in the t48 group. On the other hand, the different blood-derived formulations preserved ZO-1 staining in ARPE-19 cells with respect to t24, while showing slightly higher ZO-1 immunostaining than in cells from the t24 + 24 group. Nevertheless, cells treated with any of the blood-derived formulations showed higher ZO-1 staining delocalization in the cell cytoplasm compared to any of the control groups, and this was greater in cells treated with the supernatant formulation than with the membrane formulation (Figure 4).

## 3. Discussion

There is a large number and diversity of pathologies that affect the different structures of the eyeball, from mild and fleeting alterations to chronic and degenerative pathologies such as age-related macular degeneration, diabetic retinopathy, or macular hole in the case of pathologies affecting the posterior segment of the eye. These pathologies can lead to vision problems with pronounced and constant pain and can even result in blindness.

Most of these disorders are etiopathologically related to several processes including inflammation, apoptosis, and oxidative stress, which may individually or in combination affect cells in the posterior segment of the eye [31]. Several in vitro models have been used to attempt to mimic the various biological processes involved in the development of different vitreoretinal diseases in multiple cells of the posterior segment of the eye, including the use of blue light as an inducer of oxidative stress in retinal pigment epithelial cells [32,33].

The high complexity of ocular anatomy represents a major challenge in the development of new drug delivery systems. Over the last two decades, numerous therapeutic preparations have been developed for the treatment of multiple ocular pathologies. Although many of them have demonstrated a beneficial effect in reducing the symptomatology of these disorders, many technical and medical problems remain unresolved. Topical administration in the case of pathologies affecting the ocular surface and intravitreal administration in the case of those affecting the ocular fundus are the preferred routes of administration for the treatment of different ophthalmic conditions. In the case of vitreoretinal pathologies, current treatments focus on the application of various neuroregenerative or anti-angiogenic proteins by intravitreal infiltration; however, although some of these treatments have managed to reduce the risk of severe vision loss, they do not eliminate the risk or the problem definitively.

On the other hand, these treatments are expensive, and several infiltrations are necessary in the posterior segment of the eye to achieve the desired results, and the patient must visit the ophthalmologist several times to receive the treatment, thus increasing the cost and risk of some ocular complications [34,35].

Over the past few years, many efforts have been made to develop a drug delivery system that allows a continuous and controlled release of the drug into the retinal tissues, thereby increasing the bioavailability of the drug and, at the same time, reducing the number of visits to the ophthalmologist and therefore the risk of suffering an eye problem [5]. In addition to other features, an ideal intravitreal drug delivery system should allow the patient’s vision. In this regard, Anitua et al. have recently developed a fibrin membrane with improved optical properties that can act as a drug-delivering system [30]. The different formulations obtained from PRGF technology have been shown to have regenerative, anti-apoptotic, anti-inflammatory, and anti-oxidative stress effects [15,27,28]. In comparison to PRGF, the proteomic analysis of HK cells exposed to autologous serum has shown increased expression of inflammation, fibrosis, angiogenesis, and oxidative stress [36]. Upon the development of new formulations, these properties need to be preserved.

The present study investigates the effects of the optically enhanced membrane obtained from PRGF technology and its supernatant on an in vitro retinal model after exposure to a blue light source and its possible contribution to the protection and reversal of the cytotoxic effect of blue light on ARPE cells. These effects were also compared with those produced by a standard PRGF membrane and its supernatant.

Exposure to blue light at 500 luxes for 48 h impairs retinal cell viability and increases the production of ROS, mainly produced by mitochondria, leading to retinal damage [37]. Interestingly, however, the different PRGF product preparations (membrane and supernatant) in either formulation (PRGF or PPP-50%) reduced the phototoxic effects of blue light on ARPE cells by reducing ROS levels and improving cell viability in both protection and reversal assays.

ROS produced by oxidative stress due to blue light exposure can be released into the cytoplasm or into the mitochondrial matrix, where they can affect mitochondrial and cell viability [38]; this can be assessed using the tetrazolium salt (WST-1), which, after addition to the medium, is converted to the dye formazan by mitochondrial oxidoreductase, which is only active in viable mitochondria and living cells. The results obtained in the present study showed that the WST-1 levels of cells treated with the different PRGF-derived formulations (PRGF and PPP 50%) in both types of preparation (membrane and supernatant) were different depending on the proliferation and reversion assay. In the case of the protection assay, the different PRGF-derived formulations maintained mitochondrial activity during 48 h of blue light exposure at levels similar to the basal control (t0), although in the case of the membrane preparation, mitochondrial activity levels were slightly reduced with respect to t0. In addition, the mitochondrial activity of cells treated with the different plasma formulations was significantly higher than that of cells incubated with blue light for 48 h (t48). Similar results were obtained by Suárez-Barrio et al., where a PRGF supernatant induced an increase in mitochondrial activity and polarization in RPE cells exposed to blue light for 19 h [28]. In contrast, no significant differences in WST-1 levels were observed between the control groups and the cells treated with the different PRGF-derived formulations in the case of the reversion assay. These results are in agreement with those obtained recently by our group, wherein an oxidative stress reversal assay similar to the one used in the present work and PRGF supernatant induced a slight increase of WST-1 in ARPE-19 cells after 24 h of treatment [26]. However, the same work showed that an exposure to PRGF supernatant for an additional 24 h induced, by far, a significantly increased level of WST-1 compared to the control group. This might suggest that an increase in treatment time could induce a greater difference in mitochondrial activity between ARPE-19 cells treated with the different PRGF-derived formulations and the control group. On the other hand, in both protection and reversal assays, when WST-1 values were analyzed with respect to ROS values, the ROS/WST-1 levels of cells treated with the different PRGF-derived formulations were significantly reduced with respect to the t48 group, suggesting that treatment of cells exposed to blue light for 24 or 48 h with the different plasma formulations enhances the mitochondrial activity of the cells by lowering ROS levels more than cells exposed for 48 h to blue light. The reduction of ROS levels in ARPE-19 cells exposed to blue light after treatment with PRGF-derived formulations obtained in the present study is also in accordance with the results obtained previously by Suarez-Barrio et al. and Anitua et al. [26,28].

Under normal conditions, endogenous antioxidant compounds, including antioxidant enzymes, play a key role in maintaining oxidative balance and are the most effective in the scavenging of ROS. Oxidative stress is a cellular phenomenon or state that occurs due to a physiological imbalance between antioxidant and oxidant levels [38,39,40]. Human cells have many enzymes that are fundamental in the protection against oxidative stress, such as superoxide dismutase (SOD-1), heme oxygenase-1 (HO-1), and catalase. HO-1 is a redox-sensitive inducible stress protein that is known to protect cells against various types of stress such as oxidative stress in RPE cells [41]. In this regard, the different blood-derived formulations reduced HO-1 expression after exposure to blue light compared to the control. The latter should be in line with the lack of the need to increase antioxidant enzyme levels, as the blood-derived products kept ROS levels low. The results observed in the present study are in agreement with the results observed in a previous study, in which HO-1 levels, analyzed by immunofluorescence and Western blot, were reduced in PRGF-treated ARPE-19 cells that had previously been exposed to blue light for 24 h in a reversion assay [26]. However, the results obtained on HO-1 expression in the present study of the protection assay contrast with the results obtained by Suárez-Barrio et al., who found that PRGF treatment increased HO-1 expression in ARPE-19 cells exposed to blue light for 19 h [28]. This could be explained by the fact that HO-1 expression is time-dependent, as has been previously shown in cell culture experiments [42,43]. However, future studies should be undertaken to unravel these discordant results.

Brain epithelial barrier function depends on intercellular tight junctions and adherens junctions, including ZO-1, claudin-19, and occludin. ZO-1 is situated in the tight junction filaments of RPE cells and is considered to be one of the most important tight junction proteins in the RPE cell layer [44]. Tight junctions prevent transepithelial diffusion of solutes through the narrow spaces established between neighboring cells [45]. ZO-1 acts as a bridge between cell membrane claudins and occludins and some cytoskeletal proteins and signaling molecules, forming a protein complex involved in the regulation of epithelial cell polarization, proliferation, and differentiation [46]. Oxidative stress has been shown to disrupt RPE cell junctions and barrier integrity, promoting the pathogenesis of RPE-related diseases [47]. Oxidative stress induces in RPE cells exposed to blue light a breakdown of barrier function through the impairment of ZO-1 at tight junctions, increasing the cytoplasmic localization of ZO-1 [47,48]. The results obtained in the present study showed that the different PRGF-derived preparations increased ZO-1 staining at the cell membrane compared to cells exposed to blue light for 48 h (t48 and t24 + 24), suggesting that the plasma-derived formulations prevent ZO-1 delocalization under blue light-induced oxidative stress. These results could be explained by the presence of several proteins and growth factors in the blood-derived formulations, such as pigment epithelial growth factor (PEDF), which has been shown to reduce oxidative stress-induced disruption of RPE cell–cell junctions [49]. Furthermore, a recent study showed that treatment with PRGF supernatant induced an increase in PEDF expression in ARPE-19 cells subjected to oxidative stress by exposure to blue light, restoring the counterbalance between VEGF and PEDF expression in RPE cells [26].

In conclusion, the results obtained in the present study indicate that both the novel PRGF membrane with improved optical properties and its supernatant (M-PPP 50% and S-PPP 50%, respectively) protect and reverse blue light-induced oxidative stress in retinal pigment epithelial cells at levels similar to those of natural PRGF membrane and supernatant. Although further studies are required, these preliminary results suggest that M-PPP 50% could be used as a drug delivery system for the treatment of vitreoretinal diseases without compromising the patient’s vision.

## 4. Materials and Methods

### 4.1. Plasma Rich in Growth Factors (PRGF) Sample Collection

**Figure 5:**
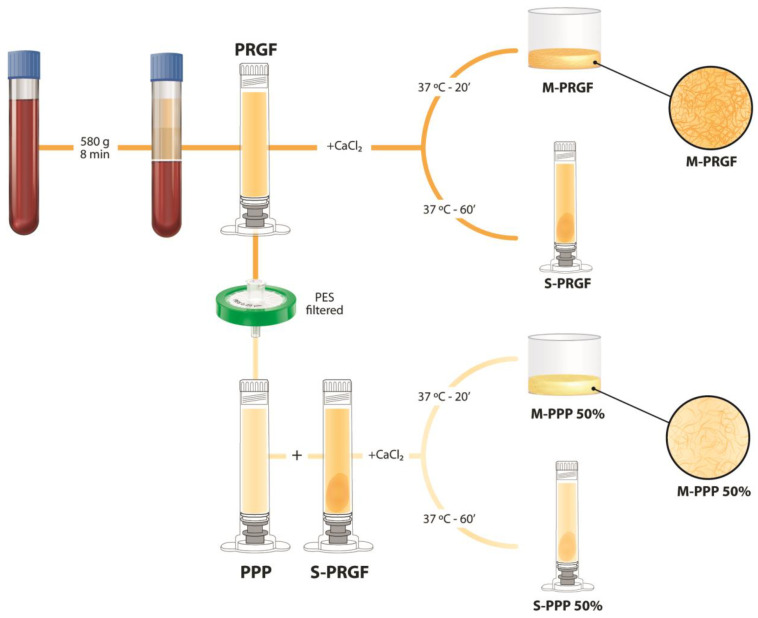
Diagram of the different formulations used to carry out this work using PRGF^®^-Endoret^®^ technology. After blood collection, it was centrifuged, and then, the entire plasma column was collected over the leukocyte layer (PRGF). The PRGF volume was then divided to obtain the different formulations used in the present study. One part was activated with calcium chloride and incubated at 37 °C for 20 min to obtain a membrane (M-PRGF) or for 60 min to obtain a supernatant (S-PRGF). Another part of the PRGF was filtered with a 0.22 µm PES filter obtaining a platelet-poor plasma (PPP) to produce the membrane with improved optical properties. It was then mixed in equal parts with S-PRGF (50% PPP and 50% S-PRGF), activated with calcium chloride, and incubated at 37 °C for 20 min to obtain a membrane (M-PPP 50%) or for 60 min to obtain a supernatant (S-PPP 50%).

To obtain the different formulations used in this study, after signing informed consent and following the principles of the Declaration of Helsinki, peripheral blood was collected from three healthy donors in 9 mL tubes with 3.8% sodium citrate. After centrifugation of the blood, the Endoret ophthalmological kit (KMU 11, BTI Biotechnology Institute, S.L., Vitoria, Alava, Spain) was used to collect the total plasma above the leukocyte layer (PRGF). Then, the total PRGF volume obtained from each donor was divided into four main groups (Figure 5): (i) M-PRGF: the PRGF was activated with PRGF activator (BTI, Vitoria, Spain) and incubated for 20 min at 37 °C to obtain a membrane in the Plasmaterm H-Plus oven (BTI, Vitoria, Spain); (ii) S-PRGF: the supernatant released from the M-PRGF after incubation for 60 min at 37 °C; (iii) M-PPP 50%: platelet free plasma (PPP) was collected after filtration of the PRGF with a 0.22 μm filter, then activated with PRGF activator, mixed 1:1 with s-PRGF, and incubated at 37 °C until clot formation; (iv) S-PPP 50%: the supernatant released from the M-PPP 50% after incubation for 60 min at 37 °C.

### 4.2. Cell Culture

The human retinal pigment epithelial cell line (ARPE-19) obtained from ATCC (Manassas, VA, USA) was seeded on the upper side of Costar transwells membrane inserts with 8 µm pores (Corning, St. Louis, MO, USA) which were pre-coated previously with bovine plasma fibronectin (Invitrogen, Thermofisher Scientific, Waltham, MA, USA). ARPE-19 cells were cultured in Dulbecco’s modified Eagle’s medium (DMEM) supplemented with 10% fetal bovine serum (FBS) and antibiotics in a humidified atmosphere of 5% CO_2_ at 37 °C for 7–8 weeks. Passage 23–27 cells were used for the experiments.

### 4.3. Blue Light-Oxidative Stress Model: Protection and Reversion Assays

To analyze the protective capacity of the different PRGF formulations tested in the present study, ARPE-19 cells cultured on the transwell chamber were treated with the different formulations as follows: for the supernatant formulations (S-PRGF and S-PPP 50%), these were diluted to 20% in DMEM/F12 culture medium and added both to the upper transwell chamber in contact with the cells and to the lower transwell chamber; for the membrane formulations (M-PRGF and M-PPP 50%), each membrane obtained from each donor was coagulated in the lower transwell chamber using a volume of 20% of the total volume of culture medium required for both upper and lower chambers and supplemented with the remaining volume of DMEM/F12 medium required to culture the ARPE-19 cells. The cells were then exposed for 48 h to a blue light LED (470 nm) at 500 lux measured with a luxometer (PCE Ibérica, Albacete, Spain). In addition, ARPE-19 cells cultured with DMEM/F12 + 1% FBS were used as a control group and analyzed before (t0) and after exposure to blue light for 48 h (t48).

On the other hand, to analyze the ability of the different formulations obtained in the present study to reverse the oxidative stress of ARPE-19 cells after exposure to blue light, the cells were cultured with DMEM/F12 + 1% FBS and incubated under the effect of blue light LEDs for 24 h to induce an initial oxidative stress (t24). The medium was then removed and the cells were treated with the usual culture medium for ARPE-19 cells (with 1% FBS and labeled t24 + 24) or with the different formulations obtained in the present study (M-PRGF, S-PRGF, M-PPP 50%, and S-PPP 50%), as mentioned above, for another 24 h while maintaining exposure to blue light. In addition, ARPE-19 cells cultured with DMEM/F12 + 1% FBS and treated with blue light for 24 h were used as a control group (t0).

### 4.4. Mitochondrial Activity and Cell Viability

Mitochondrial activity was assessed using the WST-1 (Roche, Basel, Switzerland); the stable tetrazolium salt WST-1 is cleaved to a soluble formazan dye by a complex cellular mechanism that occurs primarily at the cell surface and that is largely dependent on the glycolytic production of NAD(P)H in viable cells. Therefore, the WST-1 reagent reflects mitochondrial viability; the higher the mitochondrial dehydrogenase activity, the better the mitochondrial viability, and this is directly related to mitochondrial energy production and cell metabolic activity. The amount of formazan dye formed directly correlates to the number of metabolically active cells in the culture.

The WST-1 assay was performed by adding the reagent to wells containing treated cells and incubating at 37 °C for 30 min; finally, absorbance was measured at 450/620 nm in a multimode Synergy H microplate reader (Biotek, Vermont, VT, USA). Control wells with the same treatments, but without cells, were used for background correction. Data were represented as mean ± standard deviation (SD).

The CyQUANT cell proliferation assay (Invitrogen, Carlsbad, CA, USA) was used to analyze the cell survival in each assay (protection and reversion) achieved with each formulation developed in the present study and obtained from each donor with respect to the different controls. Briefly, after removal of the culture medium, the wells were carefully washed with phosphate-buffered saline (PBS) and the plate was frozen at −80 °C to improve the efficiency of cell lysis. Then, the plate was thawed at RT and RNase A (1.35 kU/mL) diluted in cell lysis buffer was added to each well and incubated for 1 h at RT. After that, the 2× CyQUANT GR dye/cell lysis solution was added to each well and incubated for 5 min at RT, protected from light. A multimode microplate reader (Synergy H, Biotek, VT, USA) was used to measure the fluorescence of the sample including a DNA standard curve. Data are expressed as mean ± SD in DNA concentration (ng/mL).

### 4.5. ROS Synthesis

Reactive oxygen species (ROS) production induced in ARPE-19 cells by the action of blue light was measured by a dichlorofluorescein diacetate assay (DCFDA) cellular ROS assay kit (Abcam, Cambridge, UK). After diffusion into the cell, DCFDA/H2DCFDA/DCFH-DA is converted to DCF which is a highly fluorescent compound that can be detected by fluorescence in a fluorometer. The procedure was performed according to the manufacturer’s instructions; briefly, 45 min after the end of the protection and reversion assay, DCFDA was added to the wells and incubated at 37 °C for 30 min, and finally, fluorescence (Ex/Em = 485/535 nm) was measured in a microplate fluorescence reader (Synergy H multimode reader). Data were expressed as the mean of ROS production in fluorescence units (FU) ± SD. To analyze ROS production as a function of cell viability/mitochondria and the number of cells present in each well, each individual ROS value in each well was divided by its corresponding WST-1 (ROS/WST-1) and DNA concentration (ROS/DNA) value.

### 4.6. Immunofluorescence

At the end of the different assays (protection and reversion), ARPE-19 cells were fixed with 4% formaldehyde for 10 min. Afterward, the cells were permeabilized with 0.1% Triton X-100 (Merck Life Science, Madrid, Spain) in PBS and blocked with 10% FBS in PBS. ARPE-19 cells were then incubated overnight at 4 °C simultaneously with anti-heme oxygenase-1 (anti-HO-1) (Enzo Life Sciences, Farmingdale, NY, USA) and anti-Zonula occludens-1 (ZO-1) (Invitrogen, Madrid, Spain) antibodies, both diluted 1:100 in PBS. Cells were then washed with PBS and incubated with Alexa Fluor 488 goat anti-rabbit and Alexa Fluor 594 goat anti-mouse antibodies (Molecular Probes, Thermo, ON, USA) diluted 1:100 in PBS for 1 h at room temperature. The cells were washed with PBS and finally, the cell nuclei were stained with Hoechst 33342 (Molecular Probes, Thermo, ON, USA). Images were taken for each well; three different areas from each well were photographed for each fluorophore (Hoechst 33342, Alexa Fluor 488 and, Alexa Fluor 594) using a digital camera coupled to a fluorescence microscope (Leica DM IRB). Images were processed with Image J software (v.1.52n, Wayne Rasband, NIH, Bethesda, MD, USA) to count the total number of cells and the number of HO-1 positive cells. The percentage of HO-1 positive cells in a determined area of the well was then calculated by dividing the number of HO-1 positive cells in that area by the total number of cells in the same area (stained with Hoechst) and multiplied by 100. Finally, a mean of the percentage of HO-1 positive cells derived from the 3 photographed areas of each well was obtained. The data obtained were expressed as mean ± SD of the percentage of HO-1 positive cells over the total number of ARPE-19 cells in each well.

### 4.7. Statistical Analysis

Data were expressed as mean ± SD. The Shapiro–Wilk test was used to analyze the normal distribution of each sample. Different comparisons on cell/mitochondrial viability, cell proliferation rate, ROS synthesis, and heme oxygenase-1 (HO-1) levels between the different treatment groups (M-PRGF, S-PRGF, M-PPP 50%, and S-PPP 50%) were performed using the general linear model test followed by the Bonferroni post-hoc test to analyze the statistical differences between treatment groups. In addition, ANOVA or Kruskal–Wallis tests were used to analyze the differences between the different treatment groups and the control groups used in each assay. A *p* < 0.05 was considered statistically significant. Statistical analyses were performed with the SPSS software package (v.15.0, SPSS Inc., Chicago, IL, USA).

## Data Availability

All the obtained data used to support the findings of this study are available from the corresponding author upon reasonable request.
